# Lexical associations can characterize clinical documentation trends related to palliative care and metastatic cancer

**DOI:** 10.1038/s41598-025-01828-z

**Published:** 2025-05-18

**Authors:** Hao Yuan Yang, Karthik Raghunathan, Eric Widera, Steven Z. Pantilat, Teva Brender, Timothy A. Heintz, Edie Espejo, John Boscardin, Hunter Mills, Albert Lee, Jacob Berchuck, Julien Cobert

**Affiliations:** 1 Department of Epidemiology and Biostatistics, University of California San Francisco, San Francisco, CA USA; 2 Department of Anesthesia and Perioperative Care, Duke University, Durham, NC USA; 3 Division of Geriatrics, San Francisco VA Health Care System, San Francisco, CA USA; 4 Division of Palliative Medicine, University of California San Francisco, San Francisco, CA USA; 5 Geriatrics, Palliative, and Extended Care, Veterans Affairs Medical Center, San Francisco, CA USA; 6 Department of Internal Medicine, University of California San Francisco, San Francisco, CA USA; 7 Department of Anesthesiology, Perioperative and Pain Medicine, Brigham and Women’s Hospital, Boston, MA USA; 8 Bakar Computational Health Sciences Institute, University of California San Francisco, San Francisco, CA USA; 9 Division of Oncology, Winship Cancer Institute, Emory University, Atlanta, GA USA; 10 Anesthesia Service, San Francisco VA Health Care System, 4150 Clement St Building 6, Office 206, San Francisco, CA USA; 11 Department of Anesthesia and Perioperative Care, University of California San Francisco, San Francisco, CA USA

**Keywords:** Natural language processing, Palliative care, Machine learning, Linguistics, Metastasis, Health care, Medical research

## Abstract

Palliative care is known to improve quality of life in advanced cancer. Natural language processing offers insights to how documentation around palliative care in relation to metastatic cancer has changed. We analyzed inpatient clinical notes using unsupervised language models that learn how words related to metastatic cancer (e.g. “mets”, “metastases”) and palliative care (e.g. “palliative care”, “pal care”) appear relationally and change over time. We included any note from adults hospitalized at the University of California, San Francisco system. The primary outcome was how similarly terms related to metastatic cancer and palliative care appeared in notes using a mathematical approach (cosine similarity). We used word2vec to model language numerically as vectors. Relational data between vectors was captured using cosine similarity. We performed linear regression to identify changes in these relationships of terms over time. As a sensitivity analysis, we performed the same analysis per year restricted only to patients with an ICD-9/10 diagnosis code for metastatic cancer. Metastatic cancer and palliative care terms appeared in similar contexts in clinical notes each year, suggesting a close relationship in documentation. However, over time, this relationship weakened, with these terms becoming less commonly used together as measured by cosine similarities. We found similar trends when we retrained models just on patients with a diagnosis code for metastatic cancer. Text in clinical notes offers unique insights into how medical providers document palliative care in patients with advanced malignancies and how these documentation practices evolve over time.

## Introduction

Specialty palliative care (PC) for patients with advanced cancer leads to improved quality of life and reduced symptom intensity^1^. Nationally, metastatic cancer is the most common diagnosis of patients who receive specialty PC^2^. However, despite guidance emphasizing early PC integration in metastatic cancer^3^, specialty PC remains underutilized^4^. This may reflect changing makeup of PC referrals in hospitalized settings^5^. If specialty PC is a constrained resource, then increasing referrals within one specialty may come at the expense of another, like advanced cancer. PC is also delivered in different ways in different contexts. Given, the resource constraints associated with specialty PC, much PC is provided directly by the primary clinical services or teams (so-called “primary PC”)^6^. Evolving practices and cultures of how PC is delivered are dictated by resource constraints, patient needs, hospital and clinical cultures and clinician comfort around these interventions. Thus, identifying quality PC processes is challenging.

Studies to evaluate PC processes are limited given the poor sensitivity of administrative data for ascertaining specialty PC^7^. Natural language processing (NLP) allows notes to be leveraged for novel questions about how clinicians document PC. Incorporation of note text improves PC ascertainment when structured data is lacking, allowing for improved evaluation of PC quality metrics^8^. Nonetheless, documentation of PC and quality measures around PC needs are often incomplete^9,10^ and PC ascertainment using NLP requires additional approaches beyond presence or absence of “palliative care” being mentioned in notes. Notes require certain conscious or unconscious decisions by note authors during the writing process. To the extent that language, and thus medical notes, represents thought in accordance with cognitive linguistics, the inclusion or exclusion of certain language in notes could represent whether concepts are being considered by clinicians. In patients who may stand to benefit specialty PC, like those with metastatic cancers, documentation and methods that identify language similar to PC could be used to better address gaps in care and provide insights into how PC as a concept related to metastatic cancer may be evolving.

NLP converts unstructured text data into numerical data that can be inputted into statistical models. Word embeddings represent the generation of numerical representations of words relative to other words in multidimensional space, thus maintaining important contextual relationships^11^. While these concepts may be foreign to many clinicians, they represent foundational principles in large language models (like ChatGPT) that are increasingly being used in healthcare settings. If every word in a document (or note) is converted into a unique number, then this would create a substantially large amount of features that would overwhelm many statistical modelling tasks. Word embeddings help retain contextual relationships between words and minimize the number of features before inputting them into additional models for targeted statistical tasks (like clinical risk prediction). Relational distances of these contextual numerical representations of words (word embeddings) represent ways of identifying relationships of words in clinical notes, such as how commonly they appear together or how contextually related they may be. When applied over time, embeddings also allow for the study of how language changes and evolves. For instance, word embeddings trained on New York Times articles by decade capture the temporal changes in attitudes toward women and ethnic minorities^12^. Temporal interactions of words like “palliative care” with other important concepts (e.g. metastatic disease) within notes may be captured through these contextual mappings.

We applied previously described methods^11^ from our group, using temporal word embeddings, to study semantic change over time. We traced the temporal development of concepts within notes to generate insights into the evolution of clinical documentation related to PC through language analysis. We trained neural network models on inpatient notes, one year at a time, and queried terms related to metastatic cancer and PC. We hypothesized that terms related to metastatic cancer were closely related to PC terms and this relationship increased or strengthened over time. This study was not a mixed methods study, whereby clinician authors were interviewed about their use of language and thus, is limited to documentation practices only. Nuances within documentation that represent lexical relationships beyond the simple presence or absence of a word, can inform future interventions and studies around clinical documentation practices and potentially behaviors, such as who best to target for PC interventions or alerts.

## Methods

We conducted a retrospective cohort study using de-identified notes of adults ≥ 18 years old at University of California, San Francisco (UCSF)^13^, from 2013–2020 (includes COVID-19). The primary goal of this study was to identify lexical changes within documentation at UCSF beyond presence or absence of particular terms. This study was approved by UCSF’s institutional review board (IRB 20-30590). All research was carried out in accordance with UCSF guidelines and regulations and all protocols were approved by the UCSF IRB above. Due to the retrospective nature of this study and that it was performed on de-identified data, informed consent was waived by the UCSF IRB. See Supplement for details on UCSF’s de-identified notes. We include EQUATOR guidelines to facilitate transparent reporting (STROBE as a separate file).

Some contextual information of the EHR, notes and PC services during the study period merit further discussion. Epic was formally adopted at UCSF (in its current form) in 2012–2013. Our group has noted that 2012 EHR data during the rollout of Epic can be challenging to interpret given missingness and phasing of EHR integrations and thus was not included in this study period. Inpatient PC services were available throughout this study period at UCSF but makeup of the team (e.g. inclusion of a social worker vs. chaplain) did change during the study period. Through Epic’s MyChart, UCSF notes were largely available to patients during the study period. Specific requirements around speed by which patient medical records are made available to patients online were mandated by the 21st Century CURES Act which was implemented at UCSF formally in March, 2021 which occurred after our study period. Nonetheless, many of the notes used for this study were available to patients at least within days of their writing. There were no changes to hospitals within UCSF included or excluded during the study period. Finally, patients with COVID-19 presented to UCSF as early as 2/2020 and shelter-in-place orders began the following month with important impacts on specialty PC delivery in our study population^14^.

We adopted previously described methods^11^ from our group to identify relationships of words or groups of words across clinical documents. This was assessed using a concept called “cosine similarities” which represents the “distance” of words (metastatic cancer terms and palliative care terms), not within an individual document but across all documents. Specifically, this represents the distance between the vector representations of words (word embeddings) that capture contextual elements of the words themselves. While this concept is difficult to capture compared to the presence or absence of words in general, they represent “learned” relationships by neural network models using context across a large set of documents. Stated another way, they can also be considered how likely or not one word may be replaceable by another based on the context of that word. Frequency of words present, how close they exist within notes and whether the presence of one indicates the presence or absence of another all impact the relational distances learned by neural networks.

To accomplish this, we trained word2vec models using a continuous bag of words architecture^15^, on processed words/phrases for each year. This yielded a separate NLP model for each year, allowing us to compare the same word relationships per year. See Supplement for further details on note preprocessing and word2vec models.

To develop a metastatic and PC term lexicon, we initially chose “metastatic”, “mets”, “palliative care” and “palliative”. We identified the top 50 most contextually similar words (highest cosine similarities—see below) for each term across w2v models during the study period. This identified contextually similar terms which were further refined by the authors (JC, HYY). Each added term was cross-referenced again from w2v models to further expand the lexicon. Our final lexicon is shown in Table 1. We included terms present ≥ 5 times per year (eTable 1).

**Table 1:** Refinement process of lexicon for analyzing cosine similarity in clinical notes.

Initial metastatic and PC terms chosen	Expanded lexicon of metastatic and PC terms from top 50 contextually similar terms within w2v models	Terms present in each year’s datasets	Final included list of terms after looking at most contextually similar terms
“metastatic” “mets”	“metastases”, “metastatic”, “widely_metastatic”, “metastasis”, “mets”, “widespread_metastatic”, “osseous_metastatic”, “bony_metastasis”, “metastasized”, “metastatic_deposit”, “metastatic_poorly_differentiated”, “hepatic_metastasis”, “brain_metastasis”, “bony_mets”, “metastatic_rcc”, “metastasize”, “metastatic_nsclc”, “oligometastatic”, “nodal_metastasis”, “ntrathoracic_metastatic”	“metastatic”, “widely_metastatic”, “metastasis”, “mets”, “osseous_metastatic”, “metastasized”, “metastasize”, “oligometastatic”, “nodal_metastasis”	“metastatic”, “widely_metastatic”, “metastasis”, “mets”, “osseous_metastatic”, “metastasized”, “metastasize”, “oligometastatic”, “nodal_metastasis”
“palliative” “palliative care”	“pal care” “palliate” “pall” “palliation” “pc” “palliative care” “pc consult” “palliative”	“palliate” “pall” “palliation” “pc” “palliative”	“palliate” “pall” “palliation” “palliative”

Starting from initial key terms related to metastatic disease and palliative care, the lexicon was expanded using Word2Vec models to include top 50 contextually similar terms. This expanded the term list to a larger set of terms that could represent metastatic disease and/or palliative care terms. We then queried yearly datasets to ensure presence of each word in our word2vec models. Finally, we re-expanded each term to include the top 50 contextually similar terms via models to identify whether authors were using them to convey metastatic disease or palliative care respectively. This led to the final list of terms used in the analysis. The only term removed from prior to the final lexicon was “pc” which appeared to be unrelated to the study question around palliative care and seemed to be a common acronym related to general patient care. Hence, “pc” was removed from the final list following quality control.

The primary outcome was contextual similarity or the geometric distance between the group of metastatic terms and individual target PC terms. We calculated this using cosine similarity, a commonly used metric for word embeddings, and represents the co-occurrence of words in space^16^. Outputs range from − 1 (contextually dissimilar) to + 1 (contextually similar). For instance, “SCC” and “squamous cell carcinoma” would be highly contextually similar (e.g. cosine similarity close to + 1), while words like “SCC” and an unrelated object like a “syringe” would be highly contextually dissimilar (e.g. cosine similarity close to − 1). We calculated point estimates for average cosine similarities using means and precision-weighted averages^11^. We used bootstrapping with resampling to evaluate uncertainty of point estimates^17^. Linear regression was used to determine whether base-target relationships changed over the study period.

Since running models on all inpatients would not distinguish between those with and without an actual metastatic cancer diagnosis, we also retrained models on a subset of patients with a diagnosis of metastatic cancer. This served as a sensitivity analysis and we isolated the subsets of patients with metastatic cancer International Classification of Diseases (ICD) codes-9 and -10 administrative codes. We adopted ICD-9 and ICD-10 codes for metastatic cancer from the literature. However, because our study spanned the ICD-9 to ICD-10 transition (October 2015), we used algorithms to map ICD-9 codes to ICD-10 codes using Generalized Equivalence Mapping crosswalks published by the Centers for Medicare and Medicaid Services^18^. Only codes that represented disseminated or secondary or metastatic cancers were included in the final list. See Supplement for further details on the final list of included ICD diagnosis codes (eTable 2). All w2v and linear regression models were repeated across years for this sample. All w2v models and primary and sensitivity analyses were performed using Python (version 3.8).

## Results

Across 28,600,649 inpatient adult notes from any discipline (e.g. nurses, physicians, etc.), we identified PC terms were utilized across 190,778 patients and 971,085 notes. Counts of metastatic and PC terms used annually are presented in Supplement (eTable 3). In general, counts for each term increased over time. Cosine similarities for all terms were positive (with 95% confidence intervals above zero) across each year meaning terms were more likely than chance to be co-associated with one another by note authors. The terms “metastatic” and “palliation” exhibited the highest similarity scores, with cosine similarity ranging from 0.13–0.19.

**Fig. 1:**
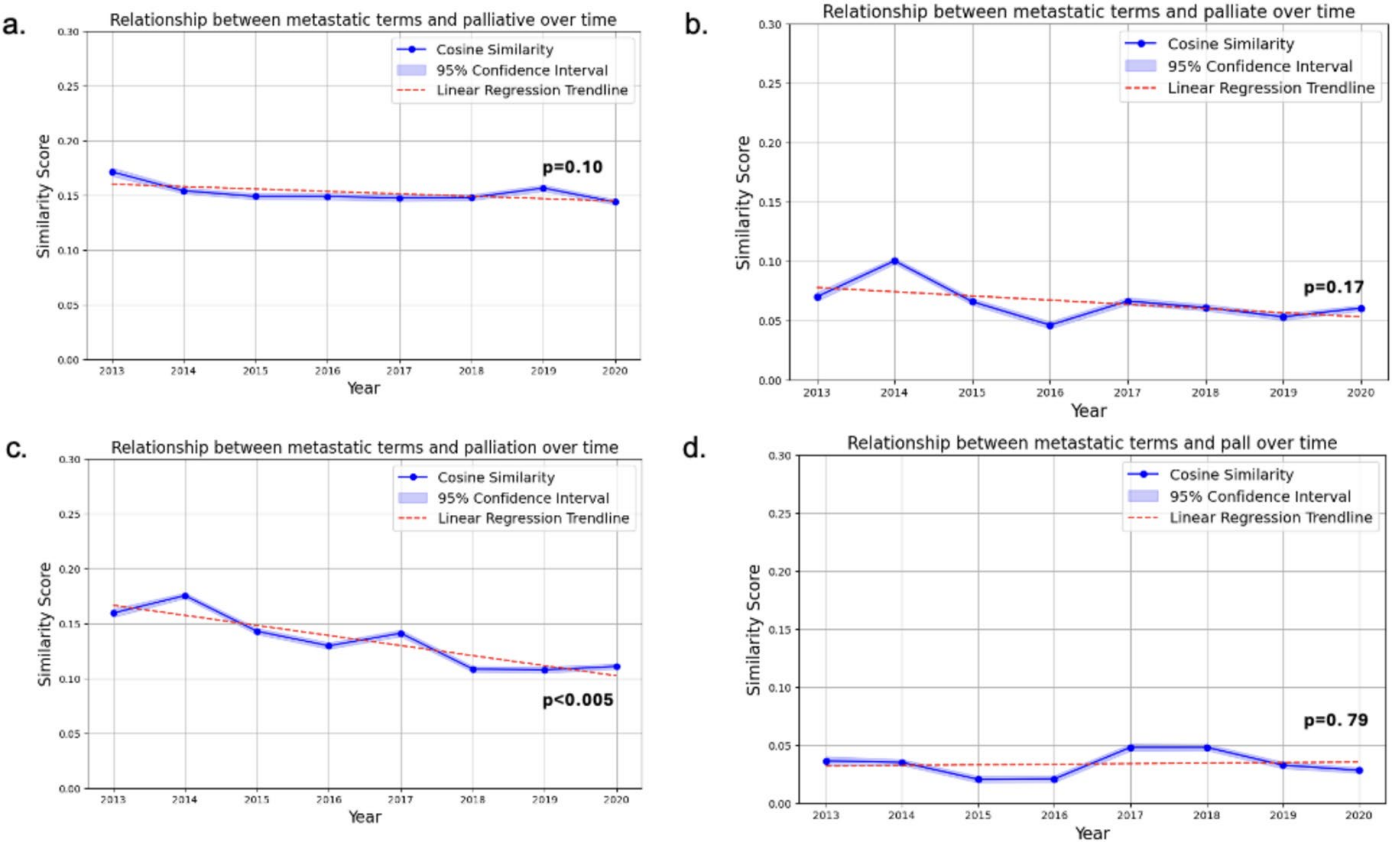
(**a**–**d**) Similarity scores of Metastatic Group and Different Palliative Terms from 2013 to 2020. Similarity scores are measured by cosine similarity (cosine angle of the mean point estimate of “metastatic” word vectors and individual palliative care target vectors). Blue lines represent the cosine similarity per year. 95% Confidence intervals for each cosine similarity value using bootstrapping with resampling, is shown vertically (and is very small for each point). Linear regression trendlines were performed for each study period. Point-wise estimates were performed to allow for the grouping of all possible “metastatic” terms. Cosine similarity ranges from − 1 (contextually dissimilar) to + 1 (contextually similar). (**a** Palliative; **b** Palliate; **c** Palliation; **d** Pall).

Cosine similarities for metastatic terms and unspecified PC terms showed a decreasing trend over time, while those for the metastatic group and “pall” remained relatively stable (Fig. 1a–d). Only metastatic terms and “palliation” showed a statistically significant decrease in cosine similarity over time. Notably, for the primary and sensitivity analyses, metastatic terms and “pall” had the smallest cosine similarities for most of the study period (these rose from 2017 to 2020), suggesting the weakest relationship among these terms. Individual base-target relationships for one example metastatic base term, are shown eFigure 2.

**Fig. 2:**
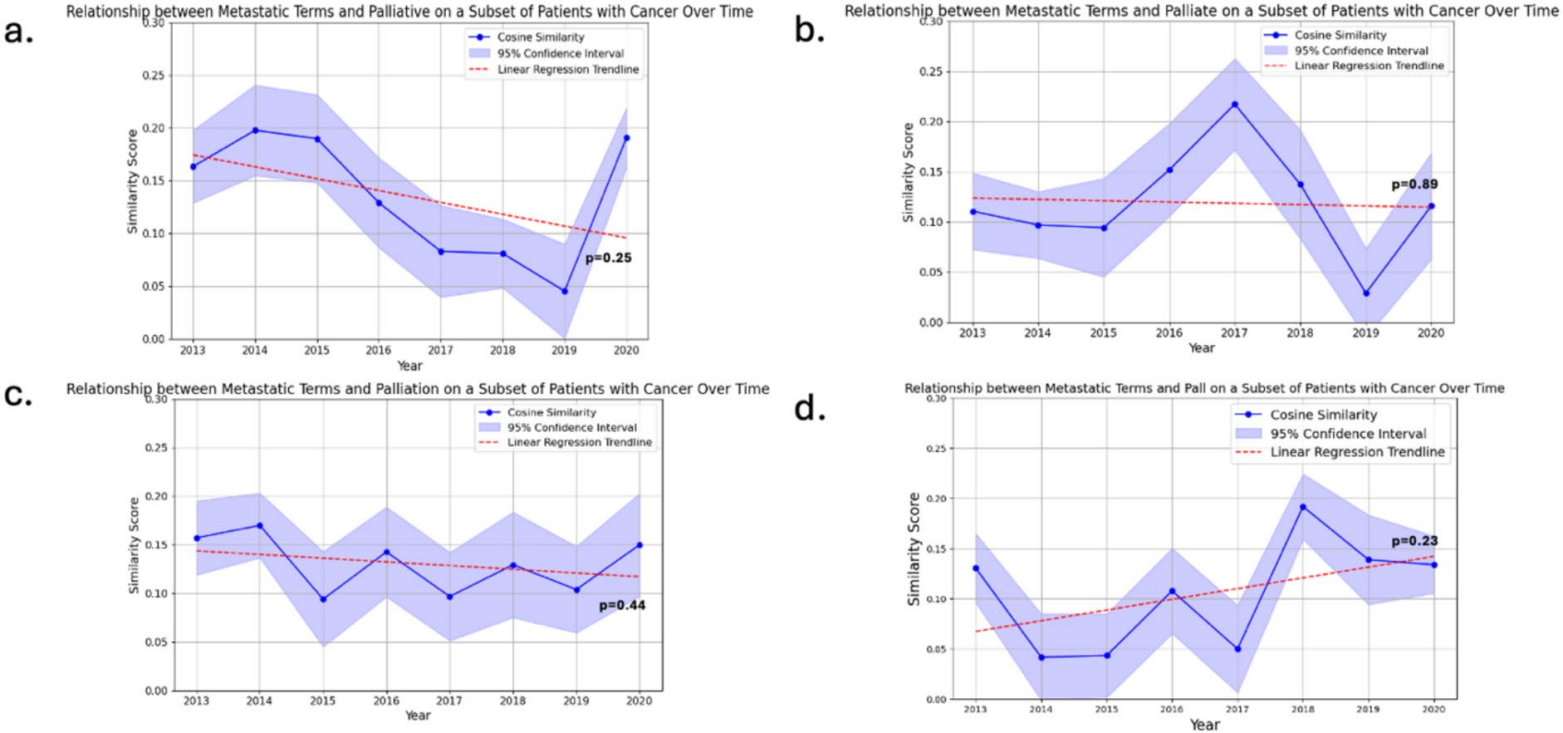
(**a**–**d**) Similarity scores of Metastatic Group and Different Palliative Terms in the Subgroup of Patients with a Metastatic Cancer Diagnosis Code from 2013 to 2020. These figures represent models only trained on notes from patients with a diagnosis code of metastatic cancer using International Classifications of Diseases codes. Similarity scores are measured by cosine similarity (cosine angle of the mean point estimate of “metastatic” word vectors and individual palliative care target vectors). Blue lines represent the cosine similarity per year. 95% Confidence intervals for each cosine similarity value using bootstrapping with resampling, is shown vertically (and is very small for each point). Linear regression trendlines were performed for each study period. Point-wise estimates were performed to allow for the grouping of all possible “metastatic” terms. Cosine similarity ranges from − 1 (contextually dissimilar) to + 1 (contextually similar). All *p*-values were > 0.05. (**a** Palliative; **b** Palliate; **c** Palliation; **d** Pall)

Patient, encounter and note counts for the patients with an ICD-code for metastatic or disseminated cancer are shown in eTable 4. The most common diagnoses included for ICD-9 were for “malignant neoplasms of ill-defined sites in the thorax” (439,824 notes across 5,833 patients), “secondary neoplasm of bone and bone marrow” (155,121 notes across 2472 patients) and “secondary malignant neoplasm of brain and spinal cord” (97,096 notes across 1258 patients). For ICD-10, the most common diagnoses included were for “secondary malignant neoplasm of other specified sites” (220,902 notes across 2,536 patients), “secondary malignant neoplasm of liver and intrahepatic bile duct” (171,148 notes across 2124 patients) and “secondary malignant neoplasm of unspecified site” (161,900 notes across 1782 patients). Cosine similarities for metastatic terms and unspecified PC terms when restricted to patients with a diagnosis code for metastatic cancer globally showed a decreasing trend over time, except for those for the metastatic group and “pall” which increased (Fig. 2a–d) but none were statistically significant (*p* > 0.2). Unlike the primary analyses, cosine similarities in the metastatic cancer subgroup increased in 2020 for “palliative” and “palliate” and slightly for “palliation”. Given the smaller corpus size (fewer notes in the metastatic cancer subgroup), confidence intervals are wider than the primary analysis and variation per year is larger.

## Discussion

We present a novel method to study the evolution of how medical providers document PC utilization in patients with advanced cancer over time. While describing the presence or absence of words for metastatic cancer and palliative care could capture broad frequencies and data about their presence in notes, they do not capture more nuanced relationships such as co-occurrences, distance between these words within notes and commonality of their contexts. To capture these ideas at a higher level (a population- or corpus-level), we leveraged neural network models using NLP. Opposite to our original hypothesis, we observed broad decreases in the relational characteristics of metastatic and PC terms, suggesting a decline in how clinicians are documenting PC generally. However, only metastatic terms and “palliation” had a statistically significant decrease in this relationship. When trained on only notes from patients with a diagnosis for metastatic cancer, the relationships became less linear and more varied but also with larger confidence intervals. Importantly, our conclusions are limited to documentation trends and lexical change and do not necessarily mean that clinicians are thinking or considering PC interventions differently over the study period. A study that explores whether clinicians are truly considering PC requires mixed methods involving interviews with note authors as they are actively thinking about patients, which is out of the scope of this study. Instead, we sought to understand relational elements of metastatic cancer and PC within notes to generate insights into how documentation itself may have evolved.

Our interest in lexical relationships is predicated on an important assumption adopted from the field of cognitive linguistics, whereby language documented by authors could represent the cognitive frameworks of those authors and thus provide insights into author behaviors^19^. Various groups have described the use of stigmatizing language within medical notes, including pejorative nouns about individuals with substance use disorders^20^ (i.e. “substance abusers”), stigmatizing adjectives^21^ and other linguistic approaches like questioning patient credibility^21^. When text is inputted into language models in an unsupervised way, language models have been shown to transmit human biases^22^. Our group has also shown that these transmissions also occur when language models are trained from medical texts instead of text from the internet^11^. Language within notes may reflect many of the conscious and unconscious subjective perceptions of the author but this study should be considered as hypothesis-generating. Future studies are required to better understand if documentation trends reflect actual clinician behaviors and considerations of PC interventions.

We acknowledge that our study results should be limited to linguistic associations and while practice patterns should not be inferred from these results, linguistic phenomena may be affected by local PC utilization and considerations. During our study period, evolving inpatient PC utilization has been reported, including increased specialty PC in patients with advanced non-cancer diagnoses relative to cancer diagnoses^5^. The decreased linguistic associations (only significant in the primary analysis for “palliation”) represents evolving documentation of how and when PC is mentioned in reference to patients with metastatic disease. Notably, any increased outpatient PC integration or adoption of primary PC delivery (PC delivered by primary medical services instead of specialty PC teams) could impact linguistic relationships if we undercaptured PC mentions in other settings for the same patients^23,24^. Future studies should pursue even more granular clinical and linguistic data (e.g. coupled to the weekly level) in order to better understand the interactions between note language and practice patterns.

Interestingly, our primary analysis demonstrated that nominalized “palliation” decreased significantly while other parts of speech like the verbalized “palliate” or adjectivized “palliative” did not. While these are closely related etymologically, the nominalized construction may reflect PC as a goal in and of itself (over say, curative therapies)^25^. While studies comparing perceptions around different variations of PC terms (e.g. palliate vs. palliative vs. palliation) have not been well-explored, stigma when the word “palliative” is used in cancer care exists^26^. It is possible that nuances between these terms could convey or represent different stigmas, perceptions, considerations or suggestions by authors^25^. Our observation that the more active constructions (e.g. “palliate”) were stable and the nominalized construction (“palliation”) decreased, could reflect such changes. However, additional research using is required to better understand how individual note authors and readers may be using and perceiving these terms differentially and how they relate to patient care.

Our study period coincides with the growth in cellular- and immunotherapies and global mortality improvements in patients with metastatic disease^27^. These could lead to changing palliative needs and hospitalization characteristics (e.g. symptoms associated with drug side effects) of such, and in turn alter how inpatient PC involvement was documented. For instance, if patients with metastatic disease were more commonly hospitalized for therapeutic side effects instead of critical illness secondary to their underlying malignancy, then language around PC needs and perceptions around when to involve specialty PC teams may have changed as well. Additionally, they could reflect shifts in culture around how note authors describe and document PC delivery and interventions more globally (e.g. PC as active interventions rather than as a goal unto itself or a subliminal shift to more outpatient PC interventions). Notably, our lexicon includes any mention of PC and did not differentiate primary vs. specialty PC which also could lead to changes in parts of speech and how the PC was actively delivered by primary vs. specialty teams. The variations in relationships between metastatic cancer and PC terms increased when restricted to training only on patients with a metastatic cancer diagnosis, suggesting a more complex relationship, meriting further study. While it is possible that further restricting training samples on particular cancers (e.g. metastatic lung cancer vs. metastatic melanoma) may reveal even more patterns unique to when and how PC is documented and considered for specific malignancies, we also anticipate wider confidence intervals. Note author interviews and qualitative methods are required to better understand how parts of speech and uses of particular PC terms may have been chosen (consciously or not) by individual authors.

Our sensitivity analyses, which retrained w2v models on only patients with a diagnosis of metastatic cancer demonstrated notable differences compared to the larger corpus of all inpatient notes. While trends in models across all patient notes largely decreased in terms of contextual similarity of metastatic cancer and PC, there was more variation and much wider CIs in the metastatic cancer only analysis. Notably, the tail end of the data in 2020 showed stronger relationships between metastatic cancer and PC. While this could be due to variations across a smaller cohort, it is also notable that COVID-19 did alter PC delivery within the UCSF system in the final year of our study period^14^. It is possible that given COVID-19-related mortality was higher in patients with metastatic cancers^28^, more patients with metastatic cancer who were also infected with COVID-19 may have had more exposure to PC services or documentation of PC by inpatient teams. While PC resources for other non-COVID-19 diseases (e.g. cancer) during the COVID-19 pandemic changed in important ways^29^, the ways clinicians *documented* PC during this time are largely unknown. One study showed increased documentation of early decision-making regarding resuscitation during the COVID pandemic^30^ but other studies around PC documentation are limited. Further studies are needed to identify how COVID-19 may have impacted documentation of PC and metastatic cancer and/or whether trends around their co-associations may have perpetuated after 2020.

Our linguistic results can help inform future studies in documentation of PC-based concepts and global considerations when using neural networks for modeling language over time. W2v, the model used for this study, learns a single fixed vector representation for each word regardless of context. Newer transformer-based^31^ models, like newer Generative Pre-trained Transformers (GPT)^32^, account for more contextual elements within a set of documents allowing for richer context-aware linguistic representations. However, using more static representations (w2v or others, like GloVe^33^ or fastText^34^) but trained yearly, could provide more opportunity to evaluate subtler “semantic drift” or changes in meaning of words or concepts longitudinally over time^35^. Notably, these approaches are somewhat more intuitive, computationally efficient and adaptable compared to transformers. Some have argued that transformers may be less useful when capturing gradual semantic changes over time compared to w2v^36^ but it remains unclear precisely which time frames would benefit from using one model versus another. Given recent digitization efforts of electronic health records (e.g. Medical Information Mart for Intensive Care [MIMIC]^37^), there are increasing opportunities to explore changes in concepts and semantics over time across different datasets (with their own unique cultures, geographies and time periods). Our group has explored differences in biases in clinical notes across different temporal and geographic datasets using these approaches^11^ and evolving considerations around concepts that are not well-defined by explicit terms, like concepts within palliative care, could be particularly notable use cases for further explorations in semantic drift. Evaluating changes in meanings or documentation over time could help describe how behaviors and practice patterns may be evolving. Importantly, when language models are trained on a large multi-year corpus of data and time is not incorporated into the architectural frameworks of the model, then relational changes between terms that do occur over time (e.g. metastatic terms and “palliation”) may be lost or oversimplified depending on the outcome of interest.

A fundamental limitation to our study is that neural networks are difficult to understand, given their non-intuitiveness. However, these techniques help us understand texts’ contextual nature and allow for linguistic analysis beyond counting words. Another important limitation is that we did not interview note authors to understand their reasons for including or excluding PC mentions nor their rationale for why they mentioned PC and metastatic cancer terms in the ways they did. We sought to leverage NLP to capture more nuanced lexical relationships *at scale* given that qualitative studies around note author language decisions are infeasible. Other limitations include possible incompleteness of our lexicon and being limited to a single hospital system. Our group has previously shown that individual hospital systems have unique cultures around language and lexical relationships are dictated by geographic place and time^11^. Hence, other note datasets are required from other systems over the same time period to determine whether broader cultural and lexical conclusions can be made. Importantly, copy-and-paste was not addressed which could confound linguistic patterns^38^. Given that the presence of mentions of metastatic cancer does not differentiate patients that actually do have metastatic cancer, we performed a sensitivity analysis on the subset of patients with an ICD code for this disease. However, ICD codes are known to have their own limitations regarding sensitivity^39^ and different metastatic cancers have very different prognoses, associated morbidities and palliative needs. Hence, given heterogeneity in our corpus, cohorts and ICD-subsets, our analyses should be interpreted broadly. Our study period included all of 2020 (through the initial COVID-19 pandemic surges) and language around care along with care delivery were undoubtedly affected by the pandemic. Results from 2020 should be taken with some caution as a result. Finally, our study was limited to the inpatient setting and future models on outpatient notes could provide further insight into changing PC practices.

## Conclusion

NLP to study changes of language within medical notes represents a novel method to better understand how authors document important interventions, like PC and how subtle changes in documentation could change over time. Unsupervised neural network models provide opportunities to leverage text features within notes to study novel questions otherwise difficult to study with structured data. Future research should determine to what extent what is documented related to PC represents how clinicians perceive, consider and use PC interventions.

## Supplementary Information

Supplementary Information.

## Data Availability

Where available data is provided within the manuscript or supplementary information files. Due to privacy concerns, UCSF’s source dataset cannot be made publicly available. However, the replication code used in the analysis is available upon request (Julien Cobert; Julien.cobert@ucsf.edu).
