# Supplementary material for: Lexical associations can characterize clinical documentation trends related to palliative care and metastatic cancer
Source: Sci Rep. 2025 May 18;15:17245. doi: 10.1038/s41598-025-01828-z (PMC12086223; doi:10.1038/s41598-025-01828-z)
Supplement: Supplementary file 1 — Supplementary Information. [file 41598_2025_1828_MOESM1_ESM.docx]

**Supplement**

**Supplemental Methods**

UCSF De-identified Clinical Data Warehouse:

The UCSF de-identified dataset has been described in detail elsewhere [1]. For UCSF notes, we used data from the De-Identified Clinical Data Warehouse (De-ID CDW) [2] which is a de-identified database copy of EHR data. This database is maintained by UCSF Information Commons using a high-performance compute cluster on Amazon Web Services clusters. Data include clinical, financial, utilization, machine-redacted clinical notes, extracted concepts and images. Machine redacted clinical notes and removal of protected health information from the UCSF De-ID CDW has been rigorously assessed. Protected health information was removed in an automated fashion using the Philter algorithm that utilizes rule-based and statistical NLP. The authors acknowledge the use of the UCSF Information Commons computational research platform, developed and supported by UCSF Bakar Computational Health Sciences Institute. De-identified research data assets were used and available through UCSF Data Access for Research (University of California, San Francisco, Academic Research systems [2022]. UCSF DeID CDW-OMOP. 2022-June. University of California, San Francisco. Dataset. Available through https://ucsf.service-now.com/ucsfit?id=ucsf_sc_cat_item&sys_id=5d5fdd2cdbec3c908a57034b8a9619c8, which is a restricted permission environment).

Preprocessing methods

For the preprocessing of textual data, we utilized the Natural Language Toolkit (NLTK) [3], version 3.8.1, to tokenize the de-identified clinical notes. The tokenization process involved breaking down text into smaller units (tokens). Following tokenization, we processed the text into n-grams using Phraser [4], a component implemented in Gensim (version 4.3.2). This method groups tokens into n-grams, which are contiguous sequences of n items from the text. The n-grams were used to train our NLP models. Our natural language processing (NLP) pipeline, including the training of word2vec (w2v) models using a continuous bag of words architecture and bootstrapping analyses, was done on Wynton, UCSF’s high-performance computational research cluster. Wynton HPC is run as a co-op with access available to all, and priority given to those who contribute funds or hardware. It currently consists of 502 nodes with 17600 cores, with each node having at least 48 GiB of RAM. The total home storage available is 770 TiB.

Bootstrapping process:

The bootstrap method was used to quantify the amount of uncertainty about our point estimate using resampled versions of our original notes dataset [5]. Standard errors estimated from this method were using to produce 95% confidence intervals for point estimates. Confidence intervals and p-values were calculated using bootstrapping. We created 50 bootstrap datasets by randomly sampling patients with replacement to preserve the correlation of notes within patient.

Unsupervised learning model (word2vec):

Word2vec converts individual n-grams to unique vectors in an n-dimensional space. Word2vec measures the closeness of vectors based on the likelihood that two vectors appear within a certain range around a word. This proximity is measured by the angle between vectors: an angle of 0 degrees indicates that the base and target pairs are identical (such as when a word matches itself), while an angle of 180 degrees shows that the pairs are contextually very different. If the angle is 90 degrees (similarity equals 1), it suggests that the contexts of each word—defined as the surrounding words in each occurrence—are equally similar and dissimilar. We used a context window of 25 tokens (words), a minimum count of 5 words, a dimensionality of 300, performed over 10 epochs for the word2vec hyperparameters.

Calculating three bases and a class of targets using precision weighted averages:

Precision-weighted averages were used to combine bases belonging to the same theme. Themes were constructed using domain knowledge. These combined base words into a single group of either words pertaining to the same theme (words pertaining to metastatic). Words belonging to themes are presented in eTable 1. In order to combine synonymous bases or bases within a class, we proposed a bootstrap statistic that uses inverse-variance weighting or precision weighting. The following was calculated


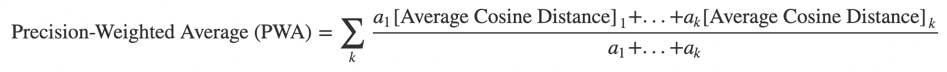


where *a_k_*=var(Average Cosine Similarity)^−1^. An average cosine similarity between a base and target pair is weighted more if there is less variability within the set of estimated cosine similarity across bootstraps. This is a weighted average of the mean cosine similarity of each of the synonymous/class bases. This essentially weights the cosine similarities between bases/targets by how variable they are.

**eTable 1**

**Presence of Terms after n-gram preprocessing in notes across years**

|  | 2010 | 2011 | 2012 | 2013 | 2014 | 2015 | 2016 | 2017 | 2018 | 2019 | 2020 |
| --- | --- | --- | --- | --- | --- | --- | --- | --- | --- | --- | --- |
| **Metastatic term** | | | | | | | | | | | |
| metastases | not present | not present | not present | not present | not present | not present | not present | not present | not present | not present | not present |
| metastatic | present | present | present | present | present | present | present | present | present | present | present |
| Widely metastatic | present | present | present | present | present | present | present | present | present | present | present |
| metastasis | present | present | present | present | present | present | present | present | present | present | present |
| mets | present | present | present | present | present | present | present | present | present | present | present |
| Widespread metastatic | present | present | not present | not present | not present | not present | not present | not present | not present | not present | not present |
| Osseous metastatic | present | present | present | present | present | present | present | present | present | present | present |
| Bony metastasis | present | present | present | present | present | present | not present | not present | not present | not present | not present |
| metastasized | present | present | present | present | present | present | present | present | present | present | present |
| Metastatic deposit | present | present | present | present | present | not present | not present | not present | not present | not present | not present |
| Metastatic poorly differentiated | present | present | present | present | present | present | not present | not present | not present | not present | not present |
| Hepatic metastasis | present | present | not present | not present | not present | not present | not present | not present | not present | not present | not present |
| Brain metastasis | present | present | not present | not present | not present | not present | not present | not present | not present | not present | not present |
| Bony mets | present | present | present | present | present | present | present | present | not present | not present | present |
| Metastatic rcc | present | present | present | present | present | not present | not present | not present | not present | not present | not present |
| metastasize | present | present | present | present | present | present | present | present | present | present | present |
| Metastatic nsclc | present | present | present | present | present | present | present | not present | not present | not present | not present |
| oligometastatic | present | present | present | present | present | present | present | present | present | present | present |
| Nodal metastasis | present | present | present | present | present | present | present | present | present | present | present |
| Ntrathoracic metastatic | not present | not present | not present | not present | not present | not present | not present | not present | not present | not present | not present |
| **Palliative term** | | | | | | | | | | | |
| pal care | not present | not present | not present | not present | not present | not present | not present | not present | not present | not present | not present |
| palliate | present | present | present | present | present | present | present | present | present | present | present |
| pall | present | present | present | present | present | present | present | present | present | present | present |
| palliation | present | present | present | present | present | present | present | present | present | present | present |
| pc | present | present | present | present | present | present | present | present | present | present | present |
| palliative care | not present | not present | not present | not present | not present | not present | not present | not present | not present | not present | not present |

The table illustrates the presence of various terms related to 'metastatic' and 'palliative' care in medical notes across years after implementing Phraser for n-gram preprocessing. The absence of certain terms ('not present') in specific years can be attributed to either their lack of usage in clinical documentation during those years or the Phraser's merging of individual terms into more frequently occurring n-grams. For example, terms like 'widespread_metastatic' and 'brain_metastasis' transition from 'present' to 'not present' as Phraser potentially combines these with other terms or they become less common in the medical discourse over time.

**eTable 2**

**International Classification of Diseases (ICD)-9 and -10 codes used to identify metastatic disease diagnoses**

The table presents the ICD codes from the ICD-9 and ICD-10 code eras (before and after 2015) adopted from the literature to identify patients with a diagnosis of metastatic or disseminated cancers. Following the ICD-9 to ICD-10 code transition, there was an increase globally in the numbers and specificity of codes relative to diseases. While no validated and accepted ICD-9 to ICD-10 code dictionary exists specifically for metastatic cancer, we used ICD-9 codes and separately ICD-10 codes from the literature and then performed an ICD crosswalk using Generalized Equivalence Mapping tools from the Centers for Medicare and Medicaid Services. This was done to ensure no ICD codes were missed for ascertaining the subset of patients with metastatic cancers.

| **ICD10** | **ICD10_Description** | **ICD9** | **ICD9_Description** |
| --- | --- | --- | --- |
| **C26** | Malignant neoplasm of other and ill-defined parts of the digestive system |  |  |
| **C39** | Malignant neoplasm of other and ill-defined sites in the respiratory system and intrathoracic organs | **165** | Malignant neoplasm of other and ill-defined sites within the respiratory system and intrathoracic organs |
| **C76** | Malignant neoplasm of other and ill-defined sites | **199.9** | Malignant neoplasm, unspecified |
| **C76.0** | Malignant neoplasm of ill-defined sites: Head, face, and neck | **199.0** | Malignant neoplasm of ill-defined sites in the head, face, and neck |
| **C76.1** | Malignant neoplasm of ill-defined sites: Thorax | **199.1** | Malignant neoplasm of ill-defined sites in the thorax |
| **C76.2** | Malignant neoplasm of ill-defined sites: Abdomen | **199.2** | Malignant neoplasm of ill-defined sites in the abdomen |
| **C76.3** | Malignant neoplasm of ill-defined sites: Pelvic | **199.3** | Malignant neoplasm of ill-defined sites in the pelvis |
| **C76.4** | Malignant neoplasm of ill-defined sites: Upper limb | **199.4** | Malignant neoplasm of ill-defined sites in the upper limb |
| **C76.5** | Malignant neoplasm of ill-defined sites: Lower limb | **199.5** | Malignant neoplasm of ill-defined sites in the lower limb |
| **C76.7** | Malignant neoplasm: Other ill-defined sites |  |  |
| **C76.8** | Malignant neoplasm: Overlapping lesion of other and ill-defined sites |  |  |
| **C77.0** | Secondary and unspecified malignant neoplasm of lymph nodes of head, face, and neck | **196** | Secondary malignant neoplasm of lymph nodes of head, face, and neck |
| **C77.1** | Secondary and unspecified malignant neoplasm of intrathoracic lymph nodes | **196.1** | Secondary malignant neoplasm of intrathoracic lymph nodes |
| **C77.2** | Secondary and unspecified malignant neoplasm of intra-abdominal lymph nodes | **196.2** | Secondary malignant neoplasm of intra-abdominal lymph nodes |
| **C77.3** | Secondary and unspecified malignant neoplasm of axilla and upper limb lymph nodes | **196.3** | Secondary malignant neoplasm of lymph nodes of axilla and upper limb |
| **C77.4** | Secondary and unspecified malignant neoplasm of inguinal and lower limb lymph nodes | **196.5** | Secondary malignant neoplasm of lymph nodes of inguinal region and lower limb |
| **C77.5** | Secondary and unspecified malignant neoplasm of intrapelvic lymph nodes | **196.6** | Secondary malignant neoplasm of intrapelvic lymph nodes |
| **C77.8** | Secondary and unspecified malignant neoplasm of lymph nodes of multiple regions | **196.8** | Secondary malignant neoplasm of lymph nodes of multiple sites |
| **C77.9** | Secondary and unspecified malignant neoplasm of lymph node, unspecified | **196.9** | Secondary malignant neoplasm of lymph nodes, site unspecified |
| **C78.0** | Secondary malignant neoplasm of lung | **197** | Secondary malignant neoplasm of lung |
| **C78.1** | Secondary malignant neoplasm of mediastinum | **197.1** | Secondary malignant neoplasm of mediastinum |
| **C78.2** | Secondary malignant neoplasm of pleura | **197.2** | Secondary malignant neoplasm of pleura |
| **C78.3** | Secondary malignant neoplasm of other and unspecified respiratory organs | **197.3** | Secondary malignant neoplasm of other respiratory organs |
| **C78.4** | Secondary malignant neoplasm of small intestine | **197.4** | Secondary malignant neoplasm of small intestine, including duodenum |
| **C78.5** | Secondary malignant neoplasm of large intestine and rectum | **197.5** | Secondary malignant neoplasm of large intestine and rectum |
| **C78.6** | Secondary malignant neoplasm of retroperitoneum and peritoneum | **197.6** | Secondary malignant neoplasm of retroperitoneum and peritoneum |
| **C78.7** | Secondary malignant neoplasm of liver and intrahepatic bile duct | **197.7** | Malignant neoplasm of liver, secondary |
| **C78.8** | Secondary malignant neoplasm of other and unspecified digestive organs | **197.8** | Secondary malignant neoplasm of other digestive organs and spleen |
| **C79.0** | Secondary malignant neoplasm of kidney and renal pelvis | **198** | Secondary malignant neoplasm of kidney |
| **C79.1** | Secondary malignant neoplasm of bladder and other urinary organs | **198.1** | Secondary malignant neoplasm of other urinary organs |
| **C79.2** | Secondary malignant neoplasm of skin | **198.2** | Secondary malignant neoplasm of skin |
| **C79.3** | Secondary malignant neoplasm of brain and cerebral meninges | **198.3** | Secondary malignant neoplasm of brain and spinal cord |
| **C79.4** | Secondary malignant neoplasm of other and unspecified parts of nervous system | **198.4** | Secondary malignant neoplasm of other parts of nervous system |
| **C79.5** | Secondary malignant neoplasm of bone and bone marrow | **198.5** | Secondary malignant neoplasm of bone and bone marrow |
| **C79.6** | Secondary malignant neoplasm of ovary | **198.6** | Secondary malignant neoplasm of ovary |
| **C79.7** | Secondary malignant neoplasm of adrenal gland | **198.7** | Secondary malignant neoplasm of adrenal gland |
| **C79.81** | Secondary malignant neoplasm of breast | **198.81** | Secondary malignant neoplasm of breast |
| **C79.82** | Secondary malignant neoplasm of genital organs | **198.82** | Secondary malignant neoplasm of genital organs |
| **C79.89** | Secondary malignant neoplasm of other specified sites | **198.89** | Secondary malignant neoplasm of other specified sites |
| **C79.9** | Secondary malignant neoplasm of unspecified site | **198.89** | Secondary malignant neoplasm of other specified sites |
| **C80.0** | Disseminated malignant neoplasm, unspecified | **199** | Disseminated malignant neoplasm without specification of site |
|  |  | **199.1** | Other malignant neoplasm without specification of site |

**eTable 3**

**Counts of preprocessed base metastatic and PC terms from the underlying DEID notes dataset**

The table presents the counts of preprocessed metastatic and palliative care terms extracted from a de-identified (DEID) notes dataset from 2013 to 2020. Each row indicates the frequency of specific terms related to metastatic and palliative care. The data shows a general increase in the mention of these terms over the years. Additionally, the total number of notes in the dataset has also grown annually.

|  | **2013** | **2014** | **2015** | **2016** | **2017** | **2018** | **2019** | **2020** |
| --- | --- | --- | --- | --- | --- | --- | --- | --- |
| **Metastatic terms** |  |  |  |  |  |  |  |  |
| **metastatic** | 60829 | 69476 | 76535 | 83784 | 95105 | 102816 | 112896 | 135696 |
| **widely_metastatic** | 1465 | 1720 | 1975 | 1530 | 1869 | 1758 | 2067 | 2732 |
| **metastasis** | 16379 | 19714 | 22489 | 25778 | 30514 | 34816 | 39684 | 50991 |
| **mets** | 17361 | 20246 | 22204 | 23477 | 26083 | 29350 | 34054 | 40881 |
| **osseous_metastatic** | 1791 | 2516 | 3748 | 4908 | 5279 | 5984 | 7376 | 10228 |
| **metastasized** | 3375 | 3819 | 4251 | 4110 | 4754 | 4907 | 5243 | 5820 |
| **metastasize** | 3513 | 3958 | 4428 | 4316 | 4986 | 5152 | 5483 | 6079 |
| **oligometastatic** | 331 | 376 | 479 | 702 | 821 | 1102 | 1523 | 2338 |
| **nodal_metastasis** | 318 | 438 | 664 | 822 | 1199 | 1567 | 1850 | 2982 |
| **Palliative care terms** |  |  |  |  |  |  |  |  |
| **Palliative** | 15751 | 18635 | 20288 | 21542 | 25727 | 30739 | 32355 | 34121 |
| **Palliation** | 1332 | 1270 | 1324 | 1388 | 1466 | 1694 | 1787 | 2043 |
| **Palliate** | 338 | 223 | 246 | 344 | 400 | 516 | 580 | 551 |
| **pall** | 46745 | 55503 | 58446 | 58580 | 62762 | 68491 | 60949 | 56304 |
| **Total Notes** | | | | | | | | |
|  | 2603276 | 2941872 | 3297848 | 3511045 | 3701520 | 3909053 | 4115113 | 4520922 |

| ICD-9 codes | Diagnosis name | patient_count | encounter_count | note_count |
| --- | --- | --- | --- | --- |
| 165 | Malignant neoplasm of other and ill-defined sites within the respiratory system and intrathoracic organs | n/a | n/a | 152 |
| 196 | Secondary malignant neoplasm of lymph nodes of head, face, and neck | n/a | n/a | 257 |
| 196.1 | Secondary malignant neoplasm of intrathoracic lymph nodes | 333 | 444 | 19022 |
| 196.2 | Secondary malignant neoplasm of intra-abdominal lymph nodes | 418 | 592 | 31572 |
| 196.3 | Secondary malignant neoplasm of lymph nodes of axilla and upper limb | 295 | 410 | 18678 |
| 196.5 | Secondary malignant neoplasm of lymph nodes of inguinal region and lower limb | 73 | 140 | 5442 |
| 196.6 | Secondary malignant neoplasm of intrapelvic lymph nodes | 145 | 243 | 12353 |
| 196.8 | Secondary malignant neoplasm of lymph nodes of multiple sites | 104 | 193 | 7009 |
| 196.9 | Secondary malignant neoplasm of lymph nodes, site unspecified | 516 | 703 | 37879 |
| 197 | Secondary malignant neoplasm of lung | 36 | 61 | 2744 |
| 197.1 | Secondary malignant neoplasm of mediastinum | 136 | 200 | 9852 |
| 197.2 | Secondary malignant neoplasm of pleura | 214 | 312 | 17182 |
| 197.3 | Secondary malignant neoplasm of other respiratory organs | 93 | 127 | 8019 |
| 197.4 | Secondary malignant neoplasm of small intestine, including duodenum | 111 | 159 | 10331 |
| 197.5 | Secondary malignant neoplasm of large intestine and rectum | 204 | 298 | 15949 |
| 197.6 | Secondary malignant neoplasm of retroperitoneum and peritoneum | 478 | 706 | 34753 |
| 197.7 | Malignant neoplasm of liver, secondary | 1179 | 1527 | 81868 |
| 197.8 | Secondary malignant neoplasm of other digestive organs and spleen | 192 | 270 | 16912 |
| 198 | Secondary malignant neoplasm of kidney | n/a | n/a | 191 |
| 198.1 | Secondary malignant neoplasm of other urinary organs | 103 | 163 | 10355 |
| 198.2 | Secondary malignant neoplasm of skin | 202 | 304 | 11909 |
| 198.3 | Secondary malignant neoplasm of brain and spinal cord | 1358 | 1861 | 97096 |
| 198.4 | Secondary malignant neoplasm of other parts of nervous system | 276 | 414 | 24354 |
| 198.5 | Secondary malignant neoplasm of bone and bone marrow | 1837 | 2472 | 155121 |
| 198.6 | Secondary malignant neoplasm of ovary | 87 | 143 | 5990 |
| 198.7 | Secondary malignant neoplasm of adrenal gland | 169 | 243 | 11121 |
| 198.81 | Secondary malignant neoplasm of breast | 41 | 73 | 3612 |
| 198.82 | Secondary malignant neoplasm of genital organs | 162 | 248 | 12539 |
| 198.89 | Secondary malignant neoplasm of other specified sites | 1044 | 1552 | 83541 |
| 199 | Disseminated malignant neoplasm without specification of site | 390 | 523 | 28936 |
| 199.1 | Malignant neoplasm of ill-defined sites in the thorax | 5833 | 7724 | 439824 |
| 199.2 | Malignant neoplasm of ill-defined sites in the abdomen | n/a | n/a | 3945 |
| 199.3 | Malignant neoplasm of ill-defined sites in the pelvis | 0 | 0 | 0 |
| 199.4 | Malignant neoplasm of ill-defined sites in the upper limb | 0 | 0 | 0 |
| 199.5 | Malignant neoplasm of ill-defined sites in the lower limb | 0 | 0 | 0 |
| ICD-10 codes |  |  |  |  |
| C76.0 | Malignant neoplasm of ill-defined sites: Head, face, and neck | 1194 | 1689 | 105128 |
| C76.1 | Malignant neoplasm of ill-defined sites: Thorax | 152 | 217 | 16091 |
| C76.2 | Malignant neoplasm of ill-defined sites: Abdomen | 111 | 158 | 10580 |
| C76.3 | Malignant neoplasm of ill-defined sites: Pelvic | 212 | 284 | 21109 |
| C76.4 | Malignant neoplasm of ill-defined sites: Upper limb | 0 | 0 | 0 |
| C76.5 | Malignant neoplasm of ill-defined sites: Lower limb | 0 | 0 | 0 |
| C76.7 | Malignant neoplasm: Other ill-defined sites | 0 | 0 | 0 |
| C76.8 | Malignant neoplasm: Overlapping lesion of other and ill-defined sites | 59 | 88 | 4965 |
| C77.0 | Secondary and unspecified malignant neoplasm of lymph nodes of head, face, and neck | 1376 | 1923 | 111210 |
| C77.1 | Secondary and unspecified malignant neoplasm of intrathoracic lymph nodes | 847 | 1134 | 59315 |
| C77.2 | Secondary and unspecified malignant neoplasm of intra-abdominal lymph nodes | 913 | 1208 | 84773 |
| C77.3 | Secondary and unspecified malignant neoplasm of axilla and upper limb lymph nodes | 534 | 708 | 34638 |
| C77.4 | Secondary and unspecified malignant neoplasm of inguinal and lower limb lymph nodes | 123 | 184 | 11324 |
| C77.5 | Secondary and unspecified malignant neoplasm of intrapelvic lymph nodes | 334 | 517 | 29174 |
| C77.8 | Secondary and unspecified malignant neoplasm of lymph nodes of multiple regions | 317 | 501 | 28235 |
| C77.9 | Secondary and unspecified malignant neoplasm of lymph node, unspecified | 1192 | 1657 | 99960 |
| C78.0 | Secondary malignant neoplasm of lung | 0 | 0 | 0 |
| C78.1 | Secondary malignant neoplasm of mediastinum | 318 | 468 | 29017 |
| C78.2 | Secondary malignant neoplasm of pleura | 588 | 876 | 51579 |
| C78.3 | Secondary malignant neoplasm of other and unspecified respiratory organs | 0 | 0 | 0 |
| C78.4 | Secondary malignant neoplasm of small intestine | 272 | 370 | 26118 |
| C78.5 | Secondary malignant neoplasm of large intestine and rectum | 407 | 569 | 35090 |
| C78.6 | Secondary malignant neoplasm of retroperitoneum and peritoneum | 1174 | 1642 | 102315 |
| C78.7 | Secondary malignant neoplasm of liver and intrahepatic bile duct | 2124 | 2811 | 171148 |
| C78.8 | Secondary malignant neoplasm of other and unspecified digestive organs | 0 | 0 | 0 |
| C79.0 | Secondary malignant neoplasm of kidney and renal pelvis | 0 | 0 | 0 |
| C79.1 | Secondary malignant neoplasm of bladder and other urinary organs | 0 | 0 | 0 |
| C79.2 | Secondary malignant neoplasm of skin | 386 | 562 | 30146 |
| C79.3 | Secondary malignant neoplasm of brain and cerebral meninges | 0 | 0 | 0 |
| C79.4 | Secondary malignant neoplasm of other and unspecified parts of nervous system | 0 | 0 | 0 |
| C79.5 | Secondary malignant neoplasm of bone and bone marrow | 0 | 0 | 0 |
| C79.6 | Secondary malignant neoplasm of ovary | 0 | 0 | 0 |
| C79.7 | Secondary malignant neoplasm of adrenal gland | 0 | 0 | 0 |
| C79.81 | Secondary malignant neoplasm of breast | 125 | 186 | 11936 |
| C79.82 | Secondary malignant neoplasm of genital organs | 379 | 558 | 31913 |
| C79.89 | Secondary malignant neoplasm of other specified sites | 2536 | 3571 | 220902 |
| C79.9 | Secondary malignant neoplasm of unspecified site | 1782 | 2527 | 161900 |
| C80.0 | Disseminated malignant neoplasm, unspecified | 294 | 436 | 25557 |

**eTable 4**

**Counts of patients, encounters and notes for each ICD code in the subset of patients with metastatic or disseminated cancer**

The table presents the counts of patients and notes used for the sensitivity analysis of w2v only trained on yearly notes of patients with a known diagnosis of metastatic cancer. Cells with <20 patients were suppressed (“n/a”) per CMS cell suppression guidance to ensure anonymity.

Abbreviations: ICD= International Classification of Diseases (-9 and -10); w2v=word2vec; CMS=Centers for Medicare and Medicaid Services; n/a=not applicable (suppressed)


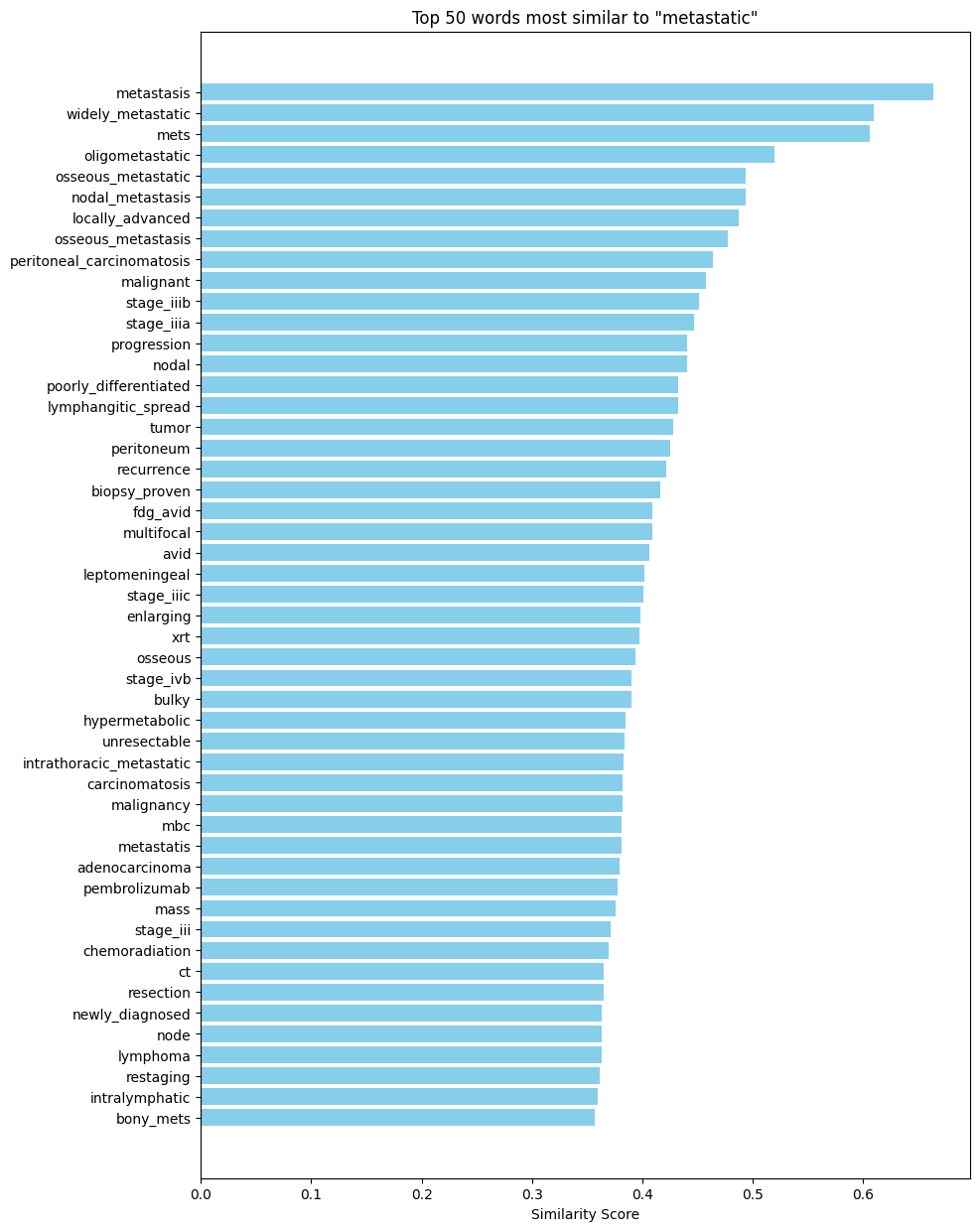


**eFigure 1 - Example of top 50 Contextually Similar Terms to 'Metastatic' from 2020**

This list displays the top 50 terms ranked by their cosine similarity scores, generated through Word2Vec models to identify words most contextually related to 'metastatic' in the dataset of the year 2020. These were used to identify other terms conveying “metastatic” disease used by note authors with similar contexts. All cosine similarities range from -1 (contextually dissimilar) to +1 (contextually similar)

**eFigure2a.**


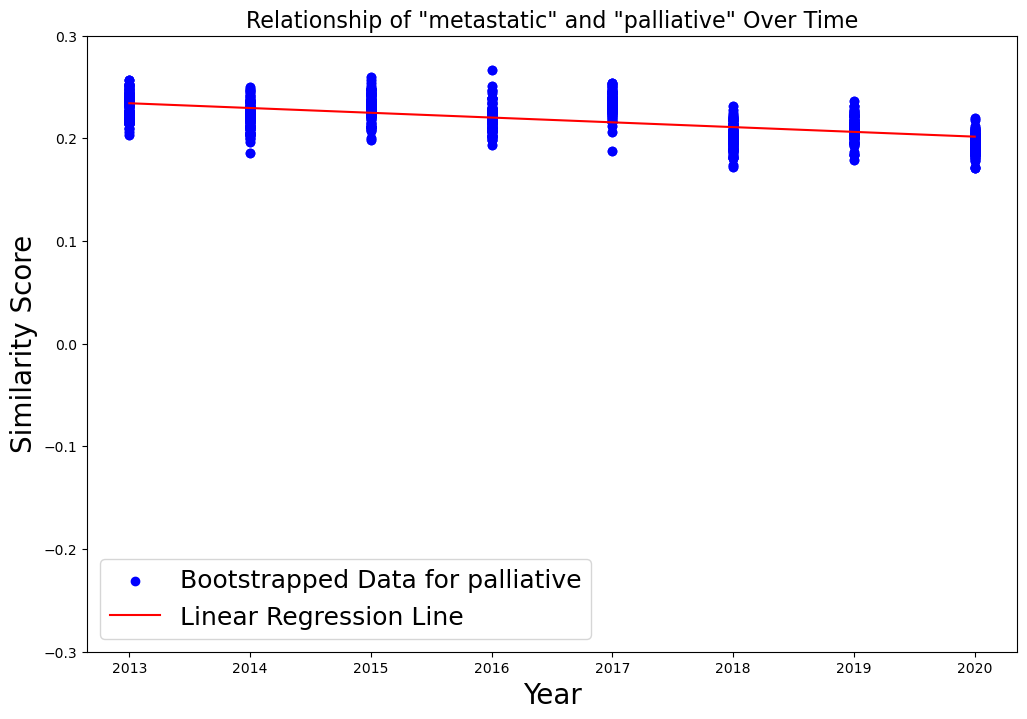


**eFigure2b.**


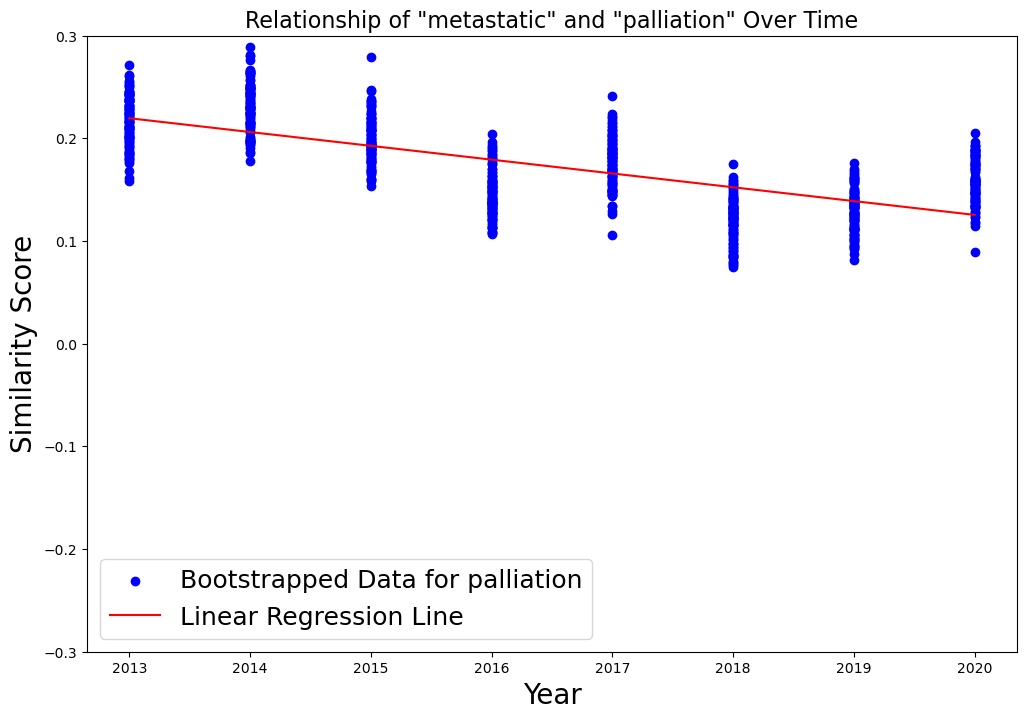


**eFigure2c.**


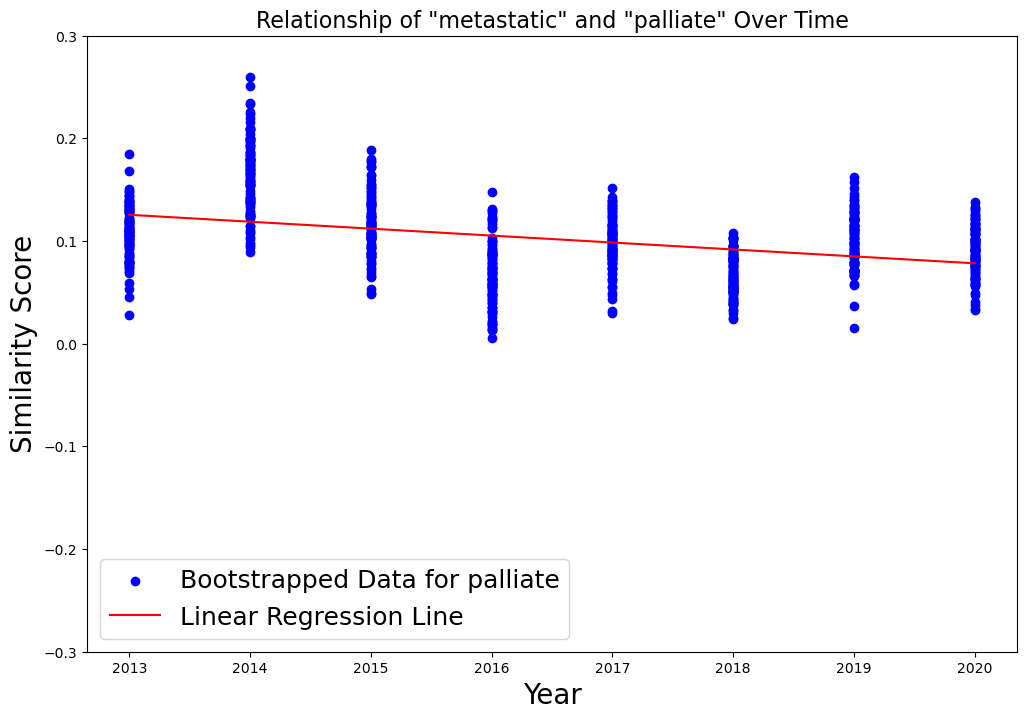


**eFigure2d.**


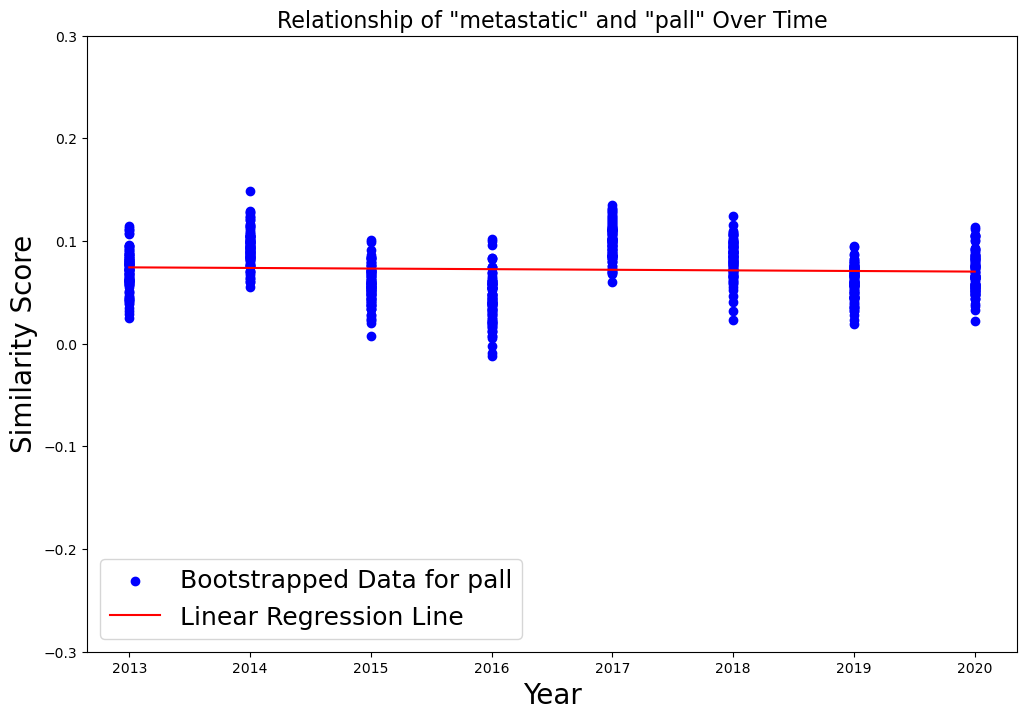


**eFigure2e.**


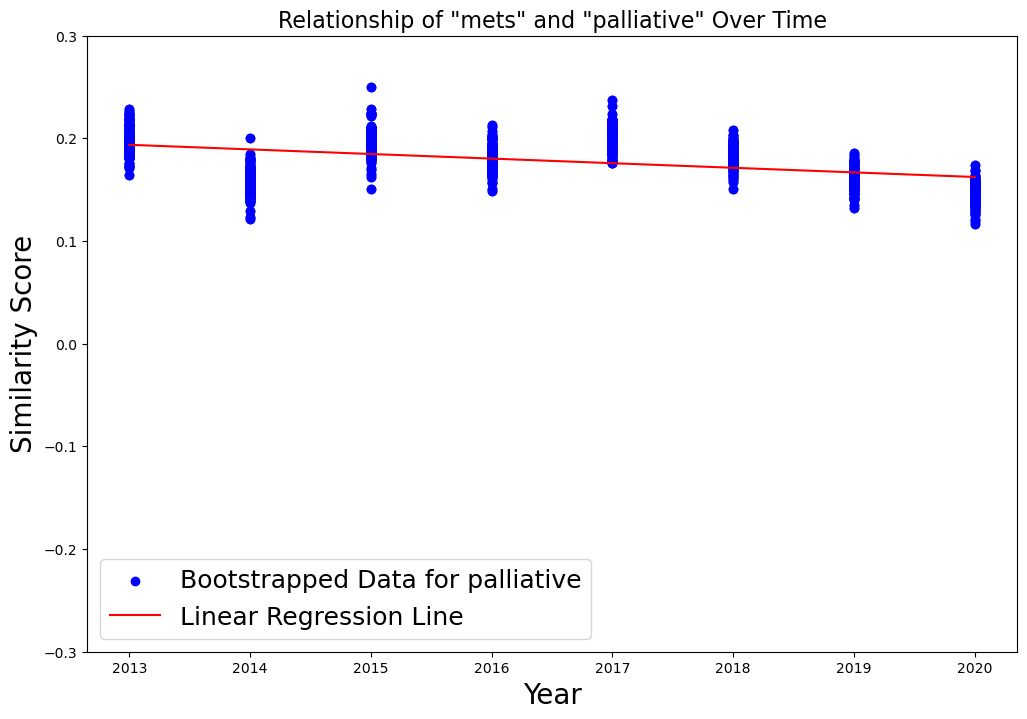


**eFigure2f.**


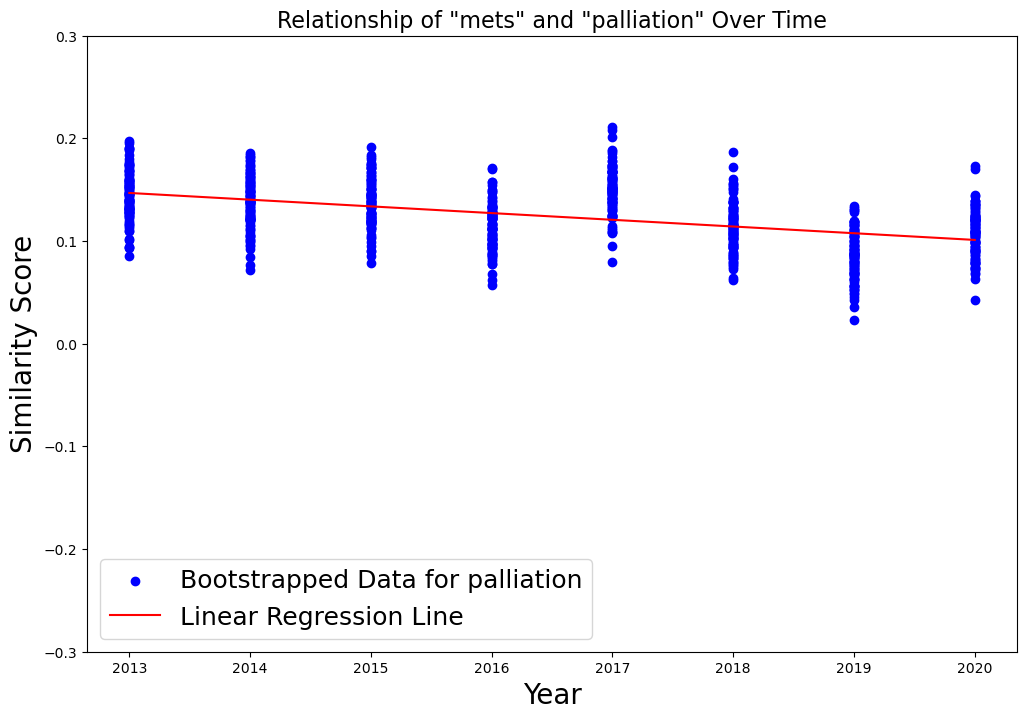


**eFigure2g.**


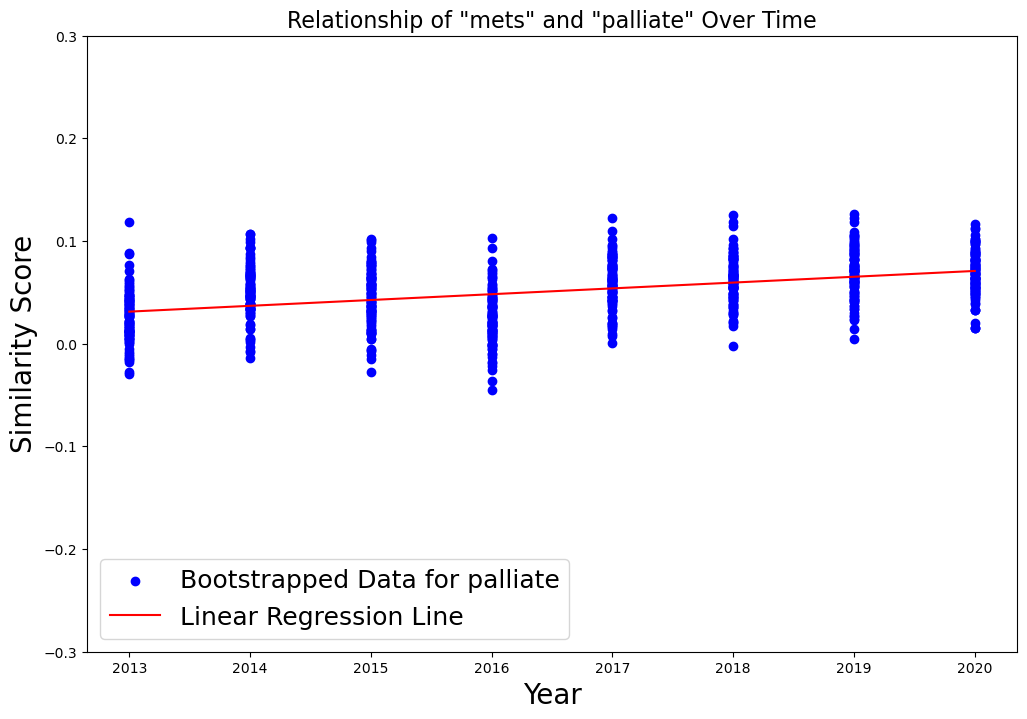


**eFigure2h.**


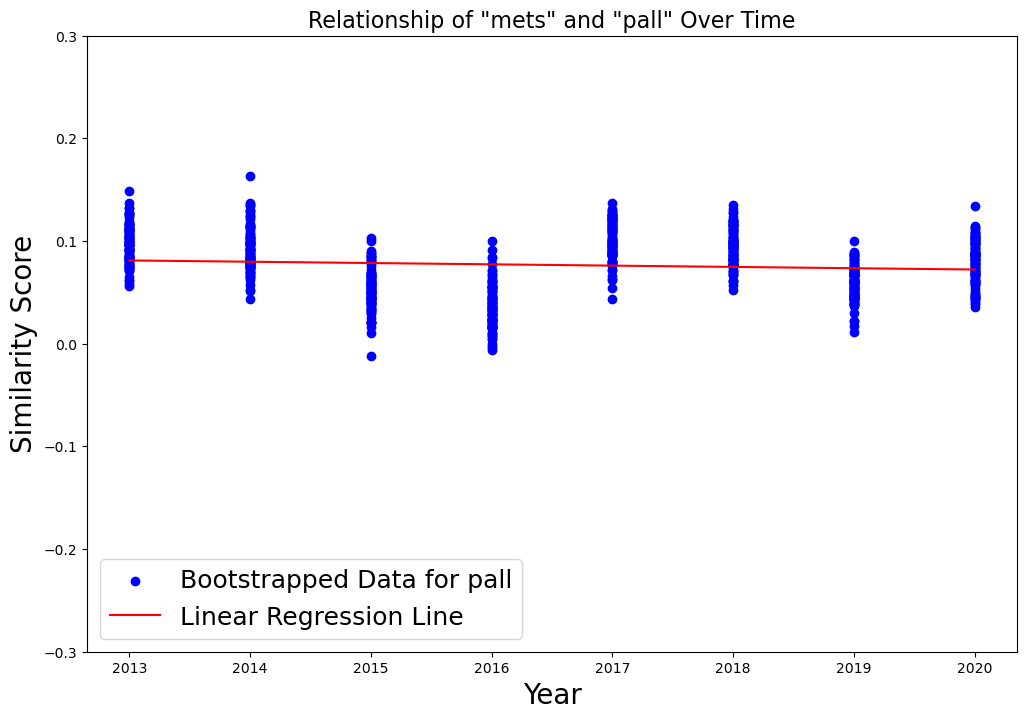


**eFigure2a-h**

**Relationship of metastatic and palliative term from 2013 to 2020**

Each graph displays the cosine similarity between specific base terms (metastatic or mets) and the target term (palliative, palliation, palliate, pall). The blue dots represent bootstrapped data points for the cosine similarity between these terms each year. Bootstrapping was employed to estimate the variability of similarity scores by resampling the data. The red line shows the trend of these relationships through linear regression analysis, indicating a slight decline over the years. Cosine similarity scores range from -1 to 1, where 1 indicates perfect similarity, 0 indicates no relationship, and -1 indicates perfect dissimilarity.

1. Radhakrishnan, L., et al., *A certified de-identification system for all clinical text documents for information extraction at scale.* JAMIA Open, 2023. **6**(3): p. ooad045.

2. Studies, H.t.G.D.-i.C.D.f.C. <https://data.ucsf.edu/research/deid-data>.

3. Bird S, K.E., Loper E., *Natural Language Processing with Python.* 1st ed. O’Reilly, 2009.

4. Řehůřek R, S.P., *Software Framework for Topic Modelling with Large Corpora In: Proceedings of the LREC 2010 Workshop on New Challenges for NLP Frameworks.* 2010. **45-50**.

5. Flamholz, Z.N., et al., *Word embeddings trained on published case reports are lightweight, effective for clinical tasks, and free of protected health information.* J Biomed Inform, 2022. **125**: p. 103971.
